# MicroRNA 101 Attenuated NSCLC Proliferation through IDH2/HIF*α* Axis Suppression in the Warburg Effect

**DOI:** 10.1155/2022/4938811

**Published:** 2022-10-18

**Authors:** Le Han, Yili Zhang, Bin Zhao, Jianren Yue, Zhenghong Chen, Guangyan Lei, Chen Huang, Wenjuan Chen

**Affiliations:** ^1^Cancer Hospital of Shaanxi Province, Affiliated to the Medical College of Xi'an Jiaotong University, Xi'an, 710061 Shaanxi, China; ^2^Department of Cell Biology and Genetics/Key Laboratory of Environment and Genes Related to Diseases, School of Basic Medical Sciences, Xi'an Jiaotong University Health Science Center, Xi'an, Shaanxi 710061, China

## Abstract

Lung cancer is the most diagnosed and deadly cancer in China. MicroRNAs are small noncoding RNA gene products that exhibit multifunctional regulation in cancer cell progressions. MiR-101 loss was illustrated in about 29% of lung cancer patients, and sophisticated mechanisms of miR-101 regulation in NSCLC are eager to be disclosed. Here, using specimens from NSCLC patients and Dural-luciferase reporter assay, we got a clue that miR-101 correlated with IDH2. MiR-101 overexpression and IDH2 deficiency both suppressed NSCLC tumor growth in mice. Moreover, in NSCLC, miR-101 suppressed IDH2 expression levels, further increased *α*-KG concentration, and finally inhibited the Warburg effect under hypoxic conditions through downregulating HIF1*α* expression by promoting HIF1*α* hydroxylation and degradation. In conclusion, miR-101 attenuated the Warburg effect and NSCLC proliferation through IDH2/HIF1*α* pathway.

## 1. Introduction

As a global health problem, lung cancer is the most diagnosed (0.82 million) and deadly cancer (0.72 million) in China in 2020 [1]. Among the lung cancer patients, about 85% belong to nonsmall cell lung cancer (NSCLC), which includes lung adenocarcinoma (LUAD), lung squamous cell carcinoma (LUSC), and large cell carcinoma histologic subtypes [2, 3]. With current anticancer therapies, we still need to put more effort into finding new ways to prolong the overall survival of NSCLC patients.

MicroRNAs are small noncoding RNA gene products with 22 nt. MicroRNAs regulate biological processes by regulating the translation and degradation of target mRNAs [4, 5]. There are four different types of miRNAs based on their location: intronic miRNAs in coding transcription units, exonic miRNAs in coding transcription units, intronic miRNAs in noncoding transcription units, and exonic miRNAs in noncoding transcription units [6]. MicroRNA 101 (miR-101) has two copies in the human genome, 1p31.3 (miR-101-1) and 9p24.1 (miR-101-2) [7]. MiR-101 exhibited downregulated expression levels in the cancers, such as lung cancer [8].

In hepatocellular carcinoma (HCC), miR-101-3p was demonstrated to suppress glycogen phosphorylase B (PYGB) expression posttranscriptionally to finally decrease cell proliferation, migration, and invasion [9]. In cardiovascular diseases, the use of mimic miR-101 could reduce COX-2 protein expression, which is highly expressed in cardiovascular diseases, by promoting mir-101 production [10]. Besides, in cancer pharmacotherapy, miR-101 could impair proteasome assembly and activity by interacting with POMP, a protein that is related to proteasome maturation [11]. Mir-101 was also reported to function as an inhibitor in autophagy [12], promoted anticancer drug toxicity [13], and inhibited postinfarct cardiac fibrosis [14].

In lung cancer patients, about 29% exhibited loss of miR-101 [15] and researchers have demonstrated that miR-101 plays an important role in NSCLC. MiR-101 is located in the middle of the regulatory pathways. One study illustrated that miR-101 could suppress DNMT3a levels and DNA methylation to inhibit lung tumorigenesis in cell lines and patient tissues [16]. In the same year, Wang et al. showed that IL-1*β* accelerated NSCLC proliferation and migration by suppressing miR-101 expression through the COX2-HIF1*α* signaling pathway, which indicated the correlation between HIF1*α* and miR-101 in NSCLC [17]. Shao et al. suggested that by attenuating miR-101 expression, exosome circ_PIP5K1A promoted NSCLC progression [18]. In 2018, Han et al. outlined that miR-101 negatively regulated NSCLC cell proliferation, invasion, and lymph node metastasis in mice and NSCLS patients. The function of miR-101 was achieved by directly downregulating zinc finger E-box binding homeobox 1 expression [19]. The newest research on miR-101 in NSCLC suggests that reduced miR-101 could promote CERS6 expression by luciferase analysis and NSCLC profile [20]. Based on the researches above, we hypothesized that there may be other mechanisms of miR-101 on NSCLC regulation.

Warburg's theory refers to metabolic reprogramming in cancer cells. Even with enough oxygen, cancer cells still were characterized by high glucose uptake rate, active glycolysis, and high content of lactic acid [21]. Although the efficiency of ATP is relatively low produced by aerobic glycolysis, it can meet the rapid supply of tumor cells due to the short overall process of glycolysis [22]. Isocitrate dehydrogenase (IDH) are enzymes that are critical in the tricarboxylic acid (TCA) cycle. IDH2 plays an indispensable role in the Warburg effect [23, 24]. IDH2 is one of the isoforms which locates in the mitochondria [25] and turns the oxidative decarboxylation of isocitrate into *α*-ketoglutarate (*α*-KG) and CO_2_ or the reverse function [26]. For example, silencing of IDH2 impaired oxidative bioenergetics, elevated reactive oxygen species (ROS) production, and promoted exaggerated mitochondrial dynamics in prostate cancer cells [27]. In addition, IDH2 could further catalyze the carboxylation of *α*-KG into citrate, leading to a reduced *α*-KG concentration [28]. *α*-KG was reported to exhibit antitumor effects through inhibition of angiogenesis in mice models [29]. *α*-KG was a substrate of the *α*-KG-dependent dioxygenases, including KDM, TET2, PHD2, and PLOD1-3, which control histone demethylation and HIF1*α*-dependent cellular signaling and collagen formation [30]. HIF1*α* is broadly expressed and correlates with poor prognosis in human cancers by regulating genes involved in glycolysis, angiogenesis, cell cycle progression, and other cellular pathways [31]. Studies demonstrated that IDH2/HIF1*α* pathway was responsible for cancer proliferation, such as cervical cancer [23] and lung cancer [26].

In this study, we made an attempt to explore the new mechanisms of miR-101 regulation in NSCLC. The dual-luciferase analysis suggested that miR-101 may target IDH2. We utilized in vivo assay to evaluate the tumor growth of NSCLC cells overexpressed with miR-101 or IDH2 deficiency. We also investigated the influence of miR-101 on the IDH2 expression levels and downstream *α*-KG concentration. Because IDH2 was critical in the Warburg effect and *α*-KG concentration was vital for HIF1*α* hydroxylation, we then evaluated the influences of miR-101 on NSCLC metabolism and HIF1*α* expression and hydroxylation. Taken together, we concluded that miR-101 attenuated NSCLC proliferation by accelerating HIF1*α* hydroxylation and degradation. These discoveries implicated the new mechanism of miR-101 in NSCLC and may provide new targets for NSCLC therapies.

## 2. Materials and Methods

### 2.1. Clinical Samples

Lung cancer tissues and corresponding adjacent tissues were obtained as mentioned before [19]. NSCLC patients enrolled without chemotherapy or radiotherapy before surgery. The surgery was conducted at the Shaanxi Provincial Cancer Hospital, affiliated to the Medical College of Xi'an Jiaotong University. All the fresh samples were collected at the time of surgery and rapidly frozen from 2019.1 to 2021.5. The details of the patient included in this study were listed in Supplementary Table 1. This study was approved by the Biomedical Ethics Committee, School of Medicine, Xi'an Jiaotong University (Approval No: 2019-622, Date: February 8, 2019).

### 2.2. Mice Model

Severe combined immunodeficiency (SCID) mice (male, 4 weeks) were purchased from Beijing Vital River Laboratory Animal Technology Co., Ltd. and then kept under aseptic conditions. The experiments were approved by the Biomedical Ethics Committee, School of Medicine, Xi'an Jiaotong University (Approval No: 2019-622, Date: February 8, 2019). 12 SCID mice were equally assigned into 4 groups randomly. Lentivirus transfected pre-miR-101-A549-luc cells (miR-101), shIDH2-A549-luc cells (shIDH2), miR-NC-A549-luc cells (miR-NC), pre-miR-101-H460-luc cells (miR-101), shIDH2-H460-luc cells (shIDH2), and miR-NC-H460-luc cells (miR-NC) were subcutaneously injected into SCID mice by 1 × 10^6^ per mouse. 150 mg/kg fluorescein was injected intraperitoneally at 2, 4, and 6 weeks after tumor cells injection. Mice were photographed with a Xenogen IVIS imaging system, the tumor sizes and volumes were analyzed, and the tumor growth curve was depicted. The nude mice were sacrificed at 8 weeks.

### 2.3. Cell Culture

We obtained 293 T cells from our lab, and we purchased A549 and H460 human NSCLC cell lines from American Type Culture Collection (Manassas, VA, USA). Cells were cultured in RPMI 1640 medium with 10% fetal bovine serum (FBS, Gibco), penicillin (100 U/mL), and streptomycin (100 U/mL) at 37°C in 5% CO2.

We cocultured A549 and H460 cells with Cycloheximide (400 *μ*M, HY-12320, MedChemExpress, USA) for 2 hours or PX-478 (10 *μ*M, HY-10231, MedChemExpress, USA) for 24 hours, or *α*-KG (1 mM, 75890, Sigma, USA) for 24 hours before subsequent examinations.

### 2.4. Real-Time Quantitative PCR (RT-qPCR)

Cells and tissues were collected and dissolved in Trizol for RNA extraction. Total RNAs were reversed into cDNA by commercial kit according to the manufacturer's instructions (SuperScript III RT, Invitrogen, USA). cDNAs were amplified by the SYBR system (Invitrogen, USA). The expression levels of mRNAs and miRNAs were calculated by the 2^-*ΔΔ*t^ method. The *β*-actin and U6 were treated as internal controls. The primers for RT-qPCR were listed in Table 1.

### 2.5. Western Blot Analysis

Tissues were homogenized with liquid nitrogen. Cells and tissues were then lysed by lysis buffer for 10 min on ice and then, centrifuged at 12,000 rpm for 15 min at 4°C. Supernatants were mixed with loading buffer and underwent SDS-PAGE to separate proteins. Proteins were then transferred into the PVDF membrane. The membranes were blocked by 5% nonfat milk in Tris-buffered saline (TBS) and then, incubated with anti-*β*-Actin (1 : 1000, ab6276, Abcam, USA), anti-HIF1*α* (1 : 500, ab179483, Abcam, USA), anti-IDH2 antibodies (1 : 500, ab131263, Abcam, USA), and anti-Hydroxy-HIF1*α* (1 : 500, 3434, Cell Signaling Technology, USA) at 4°C overnight. After being washed for 3 times by 0.5% TBST, membranes were incubated with second antibodies at a dilution of 1 : 4000 at room temperature for 2 hours then washed by 0.5% TBST for 3 times. Blots were then quantified by electrochemiluminescence and visualized by Gel Imaging System (GelDoc-It310, UVP, USA).

### 2.6. Dual-Luciferase Reporter Assay

The Dual-luciferase reporter assay was conducted as mentioned before [18]. Plasmids expressing miR-NC plus pGL3-IHD2-3′-UTR-WT, miR-NC plus pGL3-IHD2-3′-UTR-MU, miR-101 mimics plus pGL3-IHD2-3′-UTR-WT, or miR-101 mimics plus pGL3- IHD2-3′-UTR-MU were cotransfected with 293 T cells and cultured for 48 hours. Cells were lysed by lysis buffer for 15 minutes. Take 20 *μ*l cell lysate and add it to the black enzyme label plate. Add 100 *μ*l firefly luciferase reaction solution, shake the plate, and mix well to detect the activity of firefly luciferase. Add 100 *μ*l of sea kidney luciferase reaction solution, mix well with a shaking plate, and detect the activity of sea kidney luciferase.

### 2.7. Construction of Stable Cell Lines

Plasmids miR-101 mimic, miR-NC mimic, miR-101 mimic plus pUNO1-hIDH2, miR-NC mimics plus pUNO1-hIDH2, or pUNO1-hIDH2 along were cotransfected with A549 or H460 cells for 48 hours. Cells that survived 16 days of puromycin (200 *μ*g/ml) were screened for mRNA expression by RT-qPCR.

### 2.8. Flow Cytometry for ROS

1 × 106 cells were collected and washed by PBS for 3 times followed by dihydroethidium (MedChemExpress, USA) (1 *μ*M) coculture for 30 minutes. The ROS levels of cells were examined by BD FACSCanto™ II flow cytometer (BD Biosciences), which were analyzed using FlowJo 10.4 software.

### 2.9. Biochemical Analysis

The production of ATP (Cat No: BC0300, Solarbio, China), LA (Cat No: BC2235, Solarbio, China), glucose (Cat No: BC2505, Solarbio, China), and *α*-KG (Cat No: ab83431, Abcam, USA) were conducted according to the manufacturer's instructions.

### 2.10. Immunohistochemistry Analysis

Lung cancer tissues were from the Shaanxi Provincial Cancer Hospital, affiliated to the Medical College of Xi'an Jiaotong University. The study protocol was approved by the Biomedical Ethics Committee, School of Medicine, Xi'an Jiaotong University (Approval No: 2019-622, Date: February 8, 2019). Lung cancer tissues were fixed with 4% formaldehyde, embedded in paraffin, and sectioned. After the antigens were retrieved by antigen retrieval buffer, endogenous peroxidase activity was blocked by hydrogen peroxide (0.3%). The slides were stained with anti-IDH2 antibodies (1 : 200, ab131263, Abcam, USA), followed by incubation with a horseradish peroxidase-conjugated second antibody (1 : 1000, ab6721, Abcam, USA). Color was developed with diaminobenzidine and sections were counterstained with hematoxylin.

### 2.11. Statical Analysis

Statistical analysis was analyzed by GraphPad Prism 8.0 (GraphPad Software, USA) software. The significance was performed by either one-way analysis of variance (ANOVA) or unpaired, two-tailed Student *t*-test. For all tests, *p* ≤ 0.05 was considered to be statistically significant. Each experiment was repeated for 3 times, and results were presented as mean ± SD.

## 3. Results

### 3.1. miR-101 Might Regulate IDH2 in NSCLC

**Figure 1:**
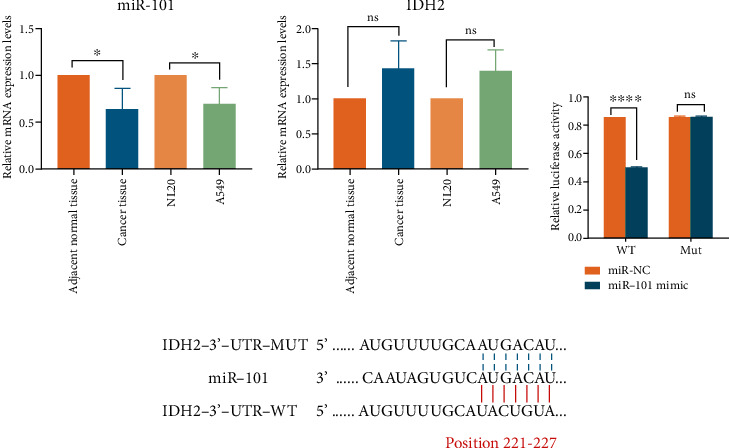
miR-101 and IDH2 expression and correlation. (a) miR-101 (left) and IDH2 mRNA (right) expression in tissues and cell lines. (b) Dural- luciferase reporter assay of miR-101 and IDH2. (c) Complemental conditions among miR-101, wild-type IDH2 and mutational IDH2. ^∗^*p* < 0.05, ^∗∗∗∗^*p* < 0.0001.

To investigate the undiscovered function of miR-101 in NSCLC, we measured mRNA expression of miR-101 using the fresh tumor samples and adjacent normal tissues collected after surgery. MiRNA-101 levels were downregulated in NSCLC tissues compared with adjacent normal tissues (Figure 1(a)) which were unanimous in a study on NSCLC [20, 32, 33]. Similar circumstances were shown in tumor A549 cells and normal lung cell lines NL20 (Figure 1(a)).

We also examined the critical protein IDH2 in cancer metabolism. Tumor tissues expressed lower levels of miR-101 than adjacent normal tissues and a higher amount of IDH2 (Figure 1(a)), which are in line with the literature that IDH2 was overexpressed in lung cancer [26, 34]. NSCLC cancer cell lines were also tested in accordance with NL20 cells. The results confirm the above conclusions (Figure 1(a)). The immunohistochemical localization of IDH2 was detected in the clinical samples, showing that IDH2 was mainly expressed in the cytoplasm (Supplementary Figure 1). We hypothesized that IDH2 might be the target of miR-101 in NSCLC. To verify the assumption, we introduced Dual-luciferase reporter assay. miR-101 were cotransfected with wild type (WT) or mutational (Mut) IDH2, and the sequences and complemental conditions were shown (Figure 1(c)). Data suggested that miR-101 significantly attenuated wild type IDH2 function without affecting mutational IDH2 (Figure 1(b)). The findings above implied that miR-101 plays a role in NSCLC by targeting wild-type IDH2.

### 3.2. MiR-101 Inhibited NSCLC Tumor Growth in Mice Models

**Figure 2:**
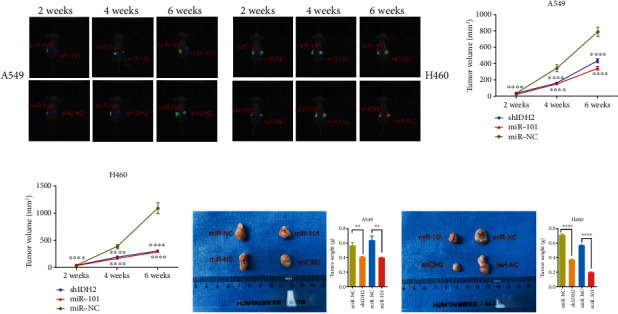
Overexpression of miR-101 or IDH2 deficiency suppressed NSCLC proliferation in mice. (a, b) SCID mice were photographed by luciferase imaging at 2 weeks, 4 weeks, and 6 weeks after transferred with A549-luc (a) and H460-luc (b). (c, d) Tumor volumes were measured by the xenografts system of A549-luc cells (c) and H460-luc cells (d). (e, f) Mice were sacrificed at 8 weeks after lung cells injection. Tumors were photographed (left) and weighted (right) of A549-luc cells (e) and H460-luc cells (f). ^∗∗^*p* < 0.01, ^∗∗∗^*p* < 0.001, ^∗∗∗∗^*p* < 0.0001.

In the previous section, we proposed that miR-101 regulates NSCLC in some aspects. To be certain, we traced the volume of tumors in SCID mice transfected with NSCLC cell lines A549-luc or H460-luc. Mice were photographed with a Xenogen IVIS imaging system every two weeks before sacrifice (Figures 2(a) and 2(b)). Before transfer, A549-luc and H460-luc cells were intervened by miR-NC or miR-101 overexpression. The volume of the tumor at every time point compared to the NC group significantly was reduced (Figures 2(c) and 2(d)). After being sacrificed at 8 weeks, tumors were photographed and weighted. Pictures and weights both indicated smaller cancer tissues (Figures 2(e) and 2(f)). Mice data make it clear that miR-101 restrains NSCLC proliferation.

Correlations between miR-101 and IDH2 were demonstrated by Dural-luciferase reporter assay (Figure 1(b)). In this part, we explored the effects of IDH2 deficiency on NSCLC proliferation in SCID mice model. Corresponding to miR-101, IDH2 deficiency in A549-luc and H460-luc cells retarded NSCLC growth by exhibiting smaller tumor volumes (Figures 2(a)–2(d)) and lighter weight (Figures 2(e) and 2(f)), which was consistent with the references that IDH2 deficiency resulted in the attenuation of lung cancer cell proliferation and tumor growth [26]. We inferred that IDH2 accelerated NSCLC proliferation and miR-101 might act through downregulated IDH2 expression.

### 3.3. MiR-101 Regulates NSCLC Metabolism through HIF1*α*

**Figure 3:**
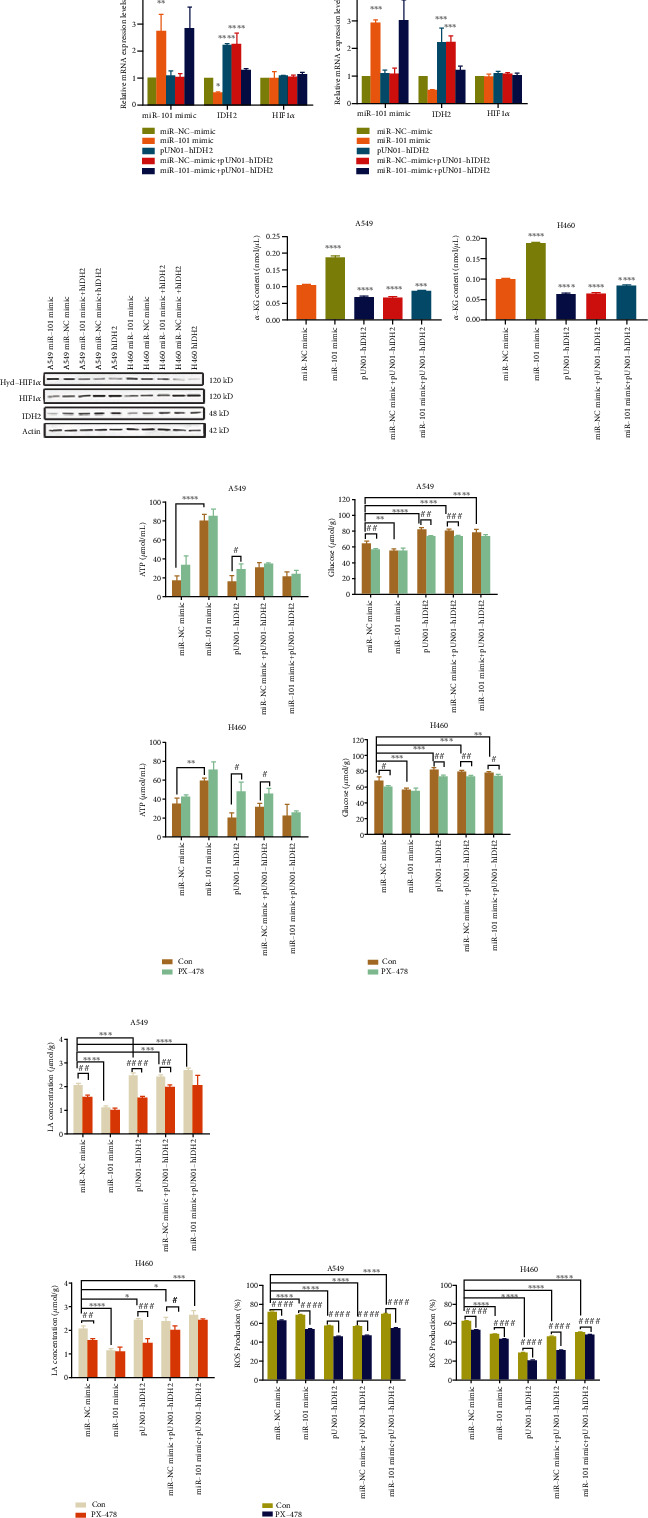
miR-101 regulated NSCLC metabolism through IDH2/HIF1*α* pathway. (a, b) The expression levels of miR-101, IDH2, and HIF1*α* were measured by RT-qPCR in stable A549 (a) and H460 (b) cells. (c) The expression levels of IDH2, HIF1*α* and HIF1*α* hydroxylation were measured by western blot analysis. (e) ATP concentrations of A549 cells (up) and H460 cells (down) were measured according to the manufacturer's instructions. (f) LA concentrations in A549 cells (up) and H460 cells (down) were measured according to the manufacturer's instructions. (g) Glucose concentrations in A549 cells (up) and H460 cells (down) were measured according to the manufacturer's instructions. (h) ROS levels of A549 cells (left) and H460 cells (right) were measured by flow cytometry analysis. ∗p < 0.05, ^∗∗^*p* < 0.01, ^∗∗∗^*p* < 0.001, ^∗∗∗∗^*p* < 0.0001.

To explore the mechanisms of miR-101 regulating NSCLC proliferation through IDH2, we overexpressed miR-101 and IDH2 in A549 and H460 cell lines separately. First, we analyzed the mutual influence between the two by RT-qPCR assay. Overexpression of IDH2 hardly influenced miR-101 levels in both A549 and H460 cells, but overexpression of miR-101 interfered with IDH2 mRNA expression in both A549 and H460 cells (Figures 3(a) and 3(b)). For further confirmation, IDH2 protein production was determined by western blot analysis. We can see that overexpression of miR-101 reduced the IDH2 expression levels compared with control (Figure 3(c) and Supplemental Figure 2 (a)). We concluded from the above data that miR-101 regulated NSCLC proliferation by modulating IDH2 expression.

To verify whether miR-101 regulated NSCLC through IDH2, because IDH2 could further catalyze the carboxylation of *α*-KG into citrate, leading to the reduced *α*-KG concentration [28], we first measured *α*-KG concentrations in A549 and H460 cells. Compared with the NC group, miR-101 promoted *α*-KG production but IDH2 alone and IDH2 plus miR-101 suppressed *α*-KG levels (Figure 3(d)), which was reported in previous studies [35–37]. These data are affiliated to prove that miR-101 regulated NSCLC through IDH2. To elucidate the relationships between miR-101 and IDH2/HIF1*α* pathway, we first measured the expression levels of HIF1*α* by RT-qPCR analysis. As data showed, HIF1*α* mRNA levels were unchanged when IDH2 overexpression with or without miR-101 (Figure 3(a)). But when we measured the protein levels of HIF1*α* and HIF1*α* hydroxylation by western blot analysis, the test results have shown that HIF1*α* was elevated with increasing IDH2 expressions and reduced hydroxylation of HIF1*α* (Figure 3(c) and Supplementary Figure 2(a)).

To disclose the mechanism of miR-101 on the Warburg effect by IDH2/HIF1*α* axis, we analyzed ATP production in NSCLC cells, glucose and lactate (LA) in cell culture medium with or without the HIF1*α* inhibitor, PX-478. MiR-101 overexpression significantly elevated ATP summation and reduced glucose uptake and LA production compared with miR-NC (Figure 3(e)–3(g)). Plus IDH2, ATP production was suppressed (Figures 3(e) and 3(f)), and glucose uptake and LA production were elevated (Figure 3(g)), which indicated that IDH2 promoted the Warburg effect in A549 and H460 cells as demonstrated in existing studies [26, 34]. Besides, PX-478 showed a distinct effect on ATP, LA and glucose concentrations (Figures 3(e)–3(g)).

Reactive oxygen species (ROS) was the byproduct of the Warburg effect which is positively related to tumor metabolism. Cancer cells do not utilize their mitochondria to the same extent and in the same way as noncancerous cells. Mitochondrial respiration is associated with the production of ROS [38]. Previous researchers discovered that IDH2 can catalyze *α*-ketoglutarate (*α*-KG) into citrate from glutamine and accelerate 2-hydroxyglutarate productions [26]. The reductive carboxylation of glutaminolysis can generate NADPH, which affiliates cellular ROS elimination.

Utilizing flow cytometry, we measured ROS production in A549 and H460 cells. MiR-101 and IDH2 along both attenuated ROS proportions which implied that miR-101 and IDH2 participated in ROS production (Figure 3(h) and Supplementary Figure 7 and 8). HIF1*α* inhibition restrained ROS levels which was in line with other studies [27]. Taken together, the data above suggested that miR-101 regulated NSCLC through IDH2/HIF1*α* pathway.

### 3.4. MiR-101 Modulated HIF1*α* Degradation

Although studies have demonstrated that IDH2/HIF1*α* pathway was indispensable in tumor growth [39–41], the mechanism under IDH2-HIF1*α* regulation kept mysterious. References demonstrated that the blocked interactions between PHD2, an *α*-KG-dependent dioxygenase [31], and HIF1*α* could inhibit the hydroxylation and degradation of HIF1*α* [42].

**Figure 4:**
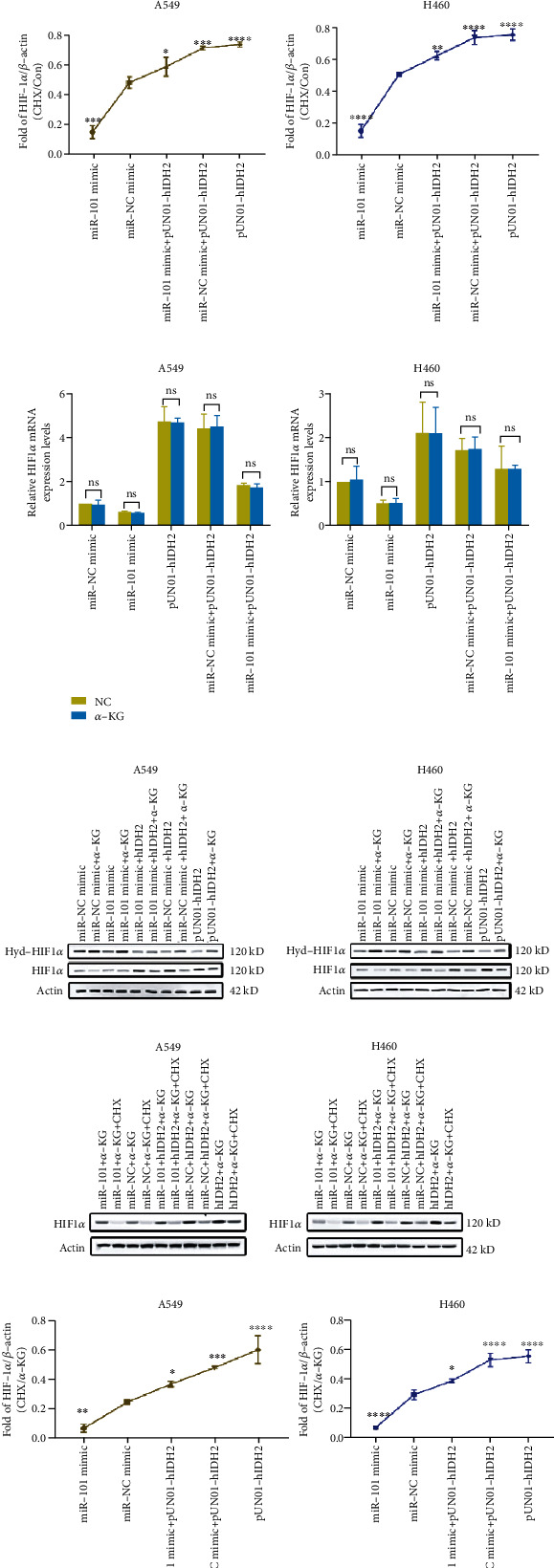
miR-101 regulated HIF1*α* hydroxylation and degradation. (a) HIF1*α* concentrations of A549 (left) and H460 (right) cells were measured by western blot after CHX treatment. (b) Statistical analysis of HIF1*α* concentrations of A549 (left) and H460 (right) cells measured by western blot after CHX treatment. (c) HIF1*α* mRNA levels were measured by RT-qPCR of A549 (left) and H460 (right) cells pretreated with *α*-KG or control. (d) The expression levels of HIF1*α* and HIF1*α* hydroxylation were measured by western blot analysis of A549 (up) and H460 (down) pretreated with *α*-KG or control. (e) HIF1*α* concentrations of A549 (left) and H460 (right) cells pretreated with *α*-KG were measured by western blot after CHX treatment. (f) Statistical analysis of HIF1*α* concentrations of A549 (left) and H460 (right) cells pretreated with *α*-KG measured by western blot after CHX treatment. ^∗^*p* < 0.05, ^∗∗^*p* < 0.01, ^∗∗∗^*p* < 0.001, ^∗∗∗∗^*p* < 0.0001.

We evaluated the protein levels of HIF1*α* after CHX treatment. Compared with the miR-NC group, miR-101 remarkably suppressed HIF1*α* production and IDH2 along accelerated HIF1*α* expression. Besides, miR-101 facilitated the IDH2-dependent HIF1*α* degradation (Figures 4(a) and 4(b)), which further demonstrated that IDH2 inhibited HIF1*α* degradation and miR-101 downregulated IDH2 functions.

As we all know, IDH2 catalyzes isocitrate into *α*-KG, which is a substrate of the *α*-KG-dependent dioxygenases, such as KDM, TET2, PHD2, and PLOD1-3, and controls histone demethylation and HIF1*α*-dependent cellular signaling and collagen formation [30]. IDH2 downregulated *α*-KG and promoted HIF1*α*-dependent signaling pathways. We appended *α*-KG to investigate deeply. We first measured HIF1*α* mRNA levels with extra *α*-KG. Results revealed no differences between the *α*-KG and control group (Figure 4(c)), which implied the HIF1*α* mRNA-independent mechanism. Subsequently, we analyzed the hydroxylation of HIF1*α* with or without *α*-KG by western blot assay. *α*-KG greatly inhibited HIF1*α* levels and promoted HIF1*α* hydroxylation (Figure 4(d) and Supplementary Figures 2(b) and (c)). After that, the changes of HIF1*α* protein were exhibited by CHX treatment. *α*-KG accelerated HIF1*α* degradation (Figures 4(e) and 4(f)). Taken together, the results above suggested that miR-101 inhibited NSCLC proliferation by accelerating HIF1*α* hydroxylation and degradation through IDH2/HIF1*α* axis.

## 4. Discussion

The Warburg effect is known to be part of metabolic reprogramming and has been discovered since the 1920s. Cancer cells utilize much more glucose than normal cells and transform glucose into lactate by aerobic glycolysis instead of metabolizing glucose by oxidative phosphorylation and shuttling the products of glycolysis into the TCA cycle [43]. IDH2/HIF1*α* axis plays a central role in the Warburg effect, which is taken advantage of by tumors for proliferation and growth [44–46].

In this study, we describe a new discovery that miR-101 attenuated NSCLC proliferation by promoting IDH2-mediated HIF1*α* hydroxylation. Firstly, to screen the possible targets of miR-101 in human NSCLC samples, we examined the mRNA levels of miR-101 which were downregulated and IDH2 which was overexpressed (Figure 1(a)) and confirmed the correlations between the two (Figure 1(b)). Data implied that miR-101 reduced IDH2 expression levels (Figures 3(a)–3(c)), and the biological function of IDH2 was retarded by miR-101 overexpression (Figure 3(d)). In vivo, the tumor-bearing mouse model illustrated the antitumor ability of miR-101 overexpression and IDH2 deficiency (Figure 2).

Further, we explored the underlying mechanisms. By inhibiting HIF1*α*, the downstream metabolisms, such as ATP (Figure 3(e)), LA (Figure 3(g)), and ROS levels (Figure 3(h)) changed as miR-101 overexpression. Besides, the hydroxylation and degradation of HIF1*α* were expedited by miR-101 overexpression in NSCLC cell lines (Figures 3(c), 4(a), and 4(b)). But the accelerations were intervened by exogenous *α*-KG (Figures 4(d)–4(f)). Together, these results indicated that miR-101 regulated NSCLC proliferation.

In this study, IDH2 played a central role in NSCLC regulation. In 2018, IDH2 was reported as a diagnostic and prognostic serum biomarker for NSCLC and high serum IDH2 levels appear to correlate with poor survival in patients with NSCLC [26]. More researches focus on IDH2 mutant. IDH2 mutations have been observed in several cancer types, including sarcomas, hematologic malignancies, colon cancer, and brain cancer [47]. Mutations in the two isocitrate dehydrogenase enzymes involved in cytoplasmic (IDH1) and mitochondrial (IDH2) conversion of alpha-ketoglutarate to D-2-hydroxyglutarate have been described as mutually exclusive in many of these cancer types. The most frequent mutations involve R132 (IDH1) and R172 (IDH2) and result in neomorphic enzyme activity. Although IDH2 (R172) mutations are associated with poorer overall prognosis in AML patients, their utility as a prognostic marker in MDS is still under debate. Additionally, IDH2 (R140) has been associated with improved overall survival in AML. IDH2 mutations have been associated with improved prognosis in gliomas. It is gratifying that an anti-IDH2 drug, enasidenib, was approved by FDA in 2017 [48, 49]. But the application was limited. The studies on wild-type of IDH2, such as this study, may provide new insights for IDH2 targeted clinical therapies.

## 5. Conclusion

The present study showed that miR-101 suppressed IDH2 expression levels, further increased *α*-KG concentration, and finally inhibited the Warburg effect by promoting HIF1*α* hydroxylation and degradation. Although we provided a valuable comprehensive landscape of miR-101 in NSCLC proliferation from many aspects, there still are some limitations in the context. The extent of miR-101 on IDH2 and whether miR-101 impacts mutated IDH2 required further studies. Next, we would dig deep and describe a more comprehensive landscape of miR-101 in the Warburg effect in NSCLC proliferation.

## Figures and Tables

**Table 1:** RT-qPCR primer sequences.

Gene	Forward (5′-3′)	Reverse ((5′-3′))
IDH2	CGCCACTATGCCGACAAAAG	ACTGCCAGATAATACGGGTCA
HIF1*α*	GAACGTCGAAAAGAAAAGTCTCG	CCTTATCAAGATGCGAACTCACA
U6	CGATACAGAGAAGATTAGCATGG	ATATGGAACGCTTCACGAA
miR-101	ACGGGCGAGCTCAGTACTGTG	CCAGTGCAGGGTCCGAGCTA
Actin	GACAGGATGCAGAAGGAGATTACT	TGATCCACATCTGCTGGAAGGT

## Data Availability

All data generated or analyzed during this study are included in this published article and its supplementary information files.

## Abbreviations

miR-101: microRNA 101
NSCLC: Nonsmall cell lung cancer
IDH2: Isocitrate dehydrogenase 2
HIF1*α*: Hypoxia-inducible factor-1*α*
LUAD: Lung adenocarcinoma
LUSC: Lung squamous cell carcinoma.

## Supplementary Materials

Supplementary Materials Supplementary Figure 1: the immunohistochemical localization of IDH2 in the clinical samples. (a) The IDH2 in a poorly differentiated squamous cell carcinoma with focal adenocarcinoma differentiation sample. (b) The IDH2 in a poorly differentiated squamous cell carcinoma with focal adenocarcinoma differentiation sample. (c) The IDH2 in an adenosquamous carcinoma. (d) The IDH2 in an adenosquamous carcinoma sample. (e) The IDH2 in a squamous carcinoma sample. (f) IDH2 in a squamous carcinoma sample. All the microscopic images were captured under 40× magnification. Supplementary Figure 2: Gray scale of each blot in western blot analysis. (a) Gray scale of IDH2, HIF1*α*, and HIF1*α* hydroxylation of A549 and H460 cells with miR-101 or IDH2 overexpression. (b) Gray scale of HIF1*α* hydroxylation (left) and HIF1*α* (right) of A549 cells pretreated with *α*-KG. (c) Gray scale of HIF1*α* hydroxylation (left) and HIF1*α* (right) of H460 cells pretreated with *α*-KG. ^∗^*p* < 0.05, ^∗∗^*p* < 0.01, _∗∗∗_*p* < 0.001, ^∗∗∗∗^*p* < 0.0001. Supplementary Figure 3: the unprocessed raw images in triplicate of Figure 3(c). The expression levels of HIF1*α* hydroxylation (a), HIF1*α* (b), IDH2 (c), and *β*-actin (d) in triplicate. Supplementary Figure 4: the unprocessed raw images in triplicate of Figure 4(a). The expression levels of HIF1*α* (a) and *β*-actin (b) of A549 cells in triplicate. The expression levels of HIF1*α* (c) and *β*-actin (d) of H460 cells in triplicate. Supplementary Figure 5: the unprocessed raw images in triplicate of Figure 4(d). The expression levels of HIF1*α* hydroxylation (a), HIF1*α* (b), and *β*-actin (c) of A549 cells in triplicate. The expression levels of HIF1*α* hydroxylation (d), HIF1*α* (e), and *β*-actin (F) of H460 cells in triplicate. Supplementary Figure 6: the unprocessed raw images in triplicate of Figure 4(e). The expression levels of HIF1*α* (a) and *β*-actin (b) of A549 cells in triplicate. The expression levels of HIF1*α* (c) and *β*-actin (d) of H460 cells in triplicate. Supplementary Figure 7: the representative dot plot and gating strategy of the FACS data for ROS estimation in A549 cells. Supplementary Figure 8: the representative dot plot and gating strategy of the FACS data for ROS estimation in H460 cells. Supplementary Table 1: the details of the patients included.
