# Supplementary material for: MicroRNA 101 Attenuated NSCLC Proliferation through IDH2/HIFα Axis Suppression in the Warburg Effect
Source: Oxid Med Cell Longev. 2022 Oct 18;2022:4938811. doi: 10.1155/2022/4938811 (PMC9596240; doi:10.1155/2022/4938811)
Supplement: Supplementary Materials — Supplementary Figure 1: the immunohistochemical localization of IDH2 in the clinical samples. (a) The IDH2 in a poorly differentiated squamous cell carcinoma with focal adenocarcinoma differentiation sample. (b) The IDH2 in a poorly differentiated squamous cell carcinoma with focal adenocarcinoma differentiation sample. (c) The IDH2 in an adenosquamous carcinoma. (d) The IDH2 in an adenosquamous carcinoma sample. (e) The IDH2 in a squamous carcinoma sample. (f) IDH2 in a squamous carcinoma sample. All the microscopic images were captured under 40× magnification. Supplementary Figure 2: Gray scale of each blot in western blot analysis. (a) Gray scale of IDH2, HIF1α, and HIF1α hydroxylation of A549 and H460 cells with miR-101 or IDH2 overexpression. (b) Gray scale of HIF1α hydroxylation (left) and HIF1α (right) of A549 cells pretreated with α-KG. (c) Gray scale of HIF1α hydroxylation (left) and HIF1α (right) of H460 cells pretreated with α-KG. ∗p < 0.05, ∗∗p < 0.01, ∗∗∗p < 0.001, ∗∗∗∗p < 0.0001. Supplementary Figure 3: the unprocessed raw images in triplicate of Figure 3(c). The expression levels of HIF1α hydroxylation (a), HIF1α (b), IDH2 (c), and β-actin (d) in triplicate. Supplementary Figure 4: the unprocessed raw images in triplicate of Figure 4(a). The expression levels of HIF1α (a) and β-actin (b) of A549 cells in triplicate. The expression levels of HIF1α (c) and β-actin (d) of H460 cells in triplicate. Supplementary Figure 5: the unprocessed raw images in triplicate of Figure 4(d). The expression levels of HIF1α hydroxylation (a), HIF1α (b), and β-actin (c) of A549 cells in triplicate. The expression levels of HIF1α hydroxylation (d), HIF1α (e), and β-actin (F) of H460 cells in triplicate. Supplementary Figure 6: the unprocessed raw images in triplicate of Figure 4(e). The expression levels of HIF1α (a) and β-actin (b) of A549 cells in triplicate. The expression levels of HIF1α (c) and β-actin (d) of H460 cells in triplicate. Supplementary Figure 7: the repre [file 4938811.f1.doc]

**
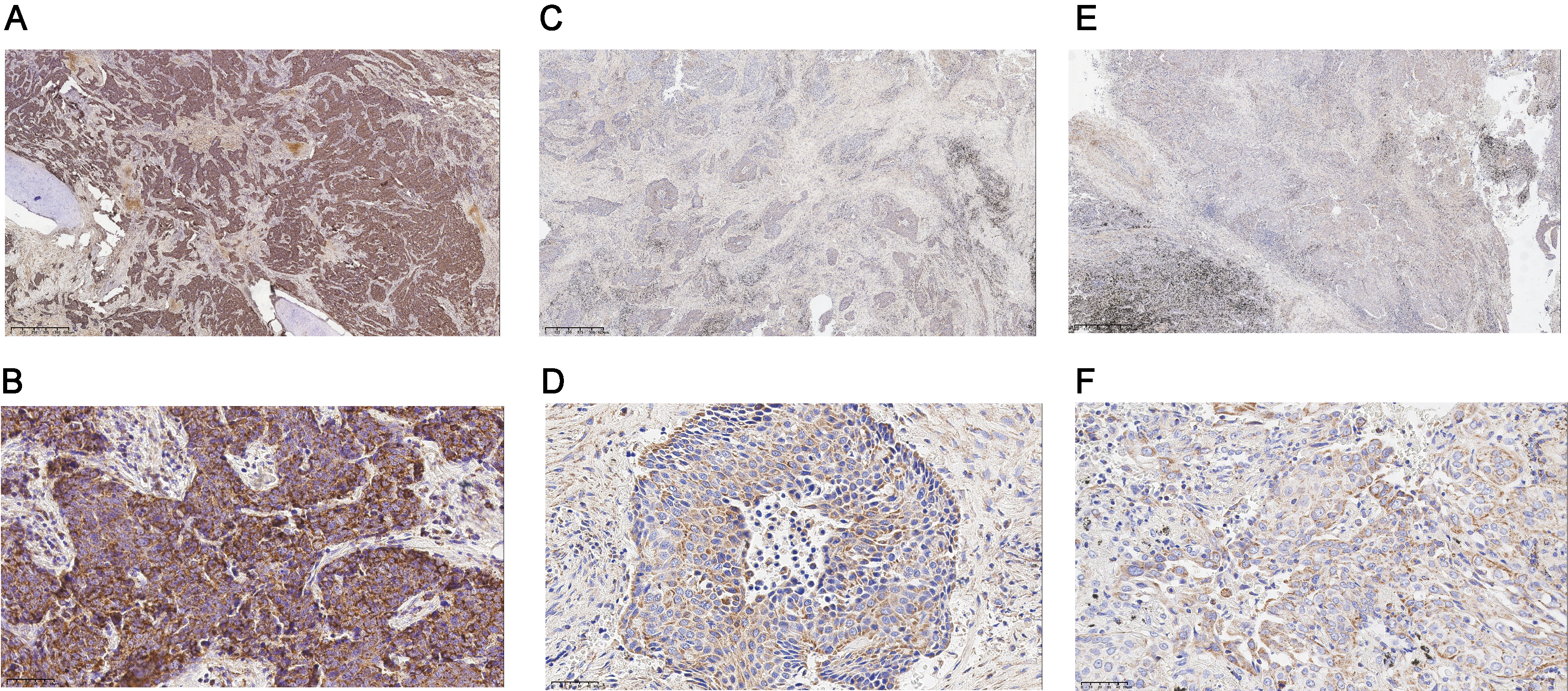
**

**Supplementary Figure 1**


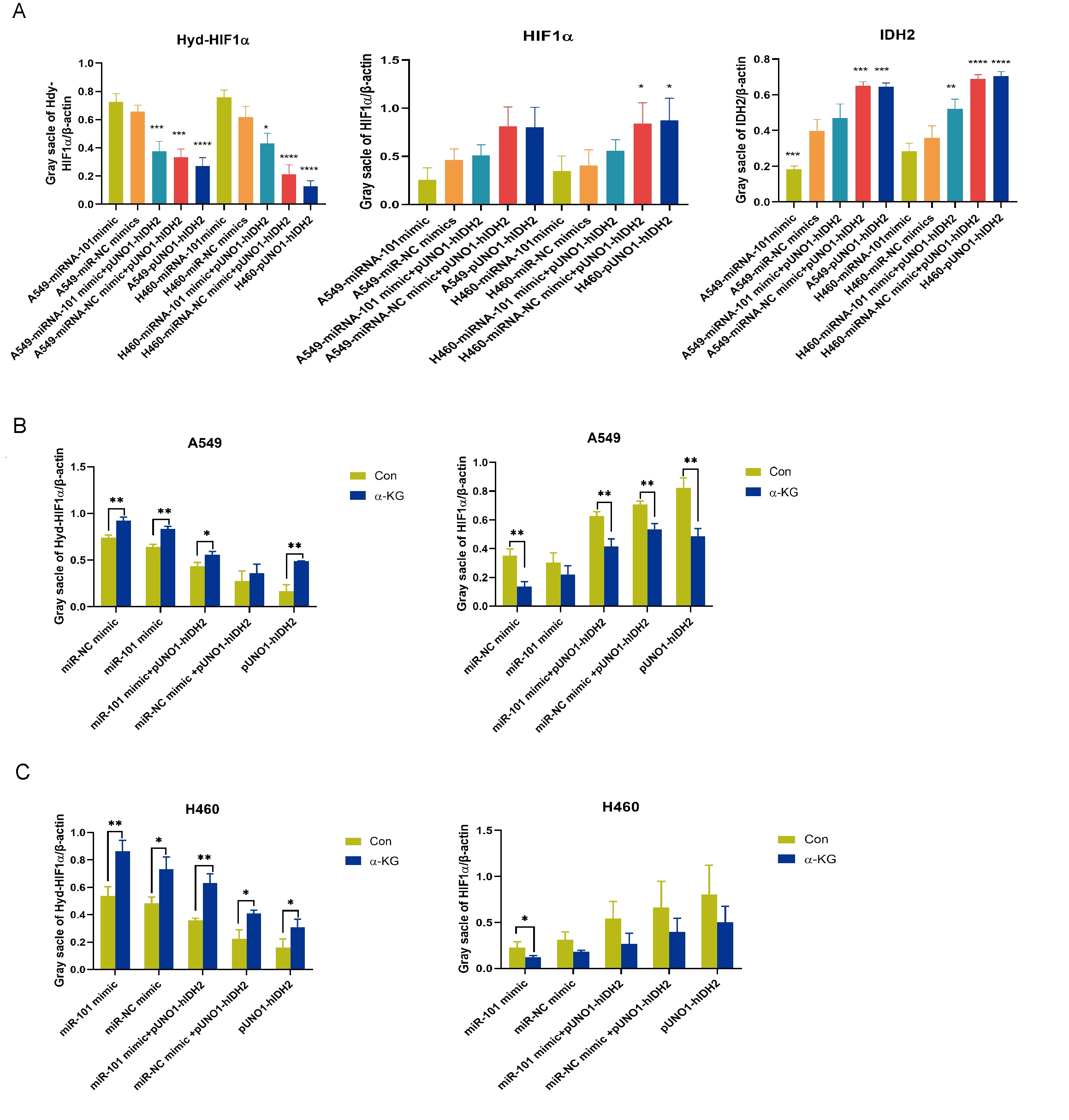


**Supplementary** **Figure 2**

**
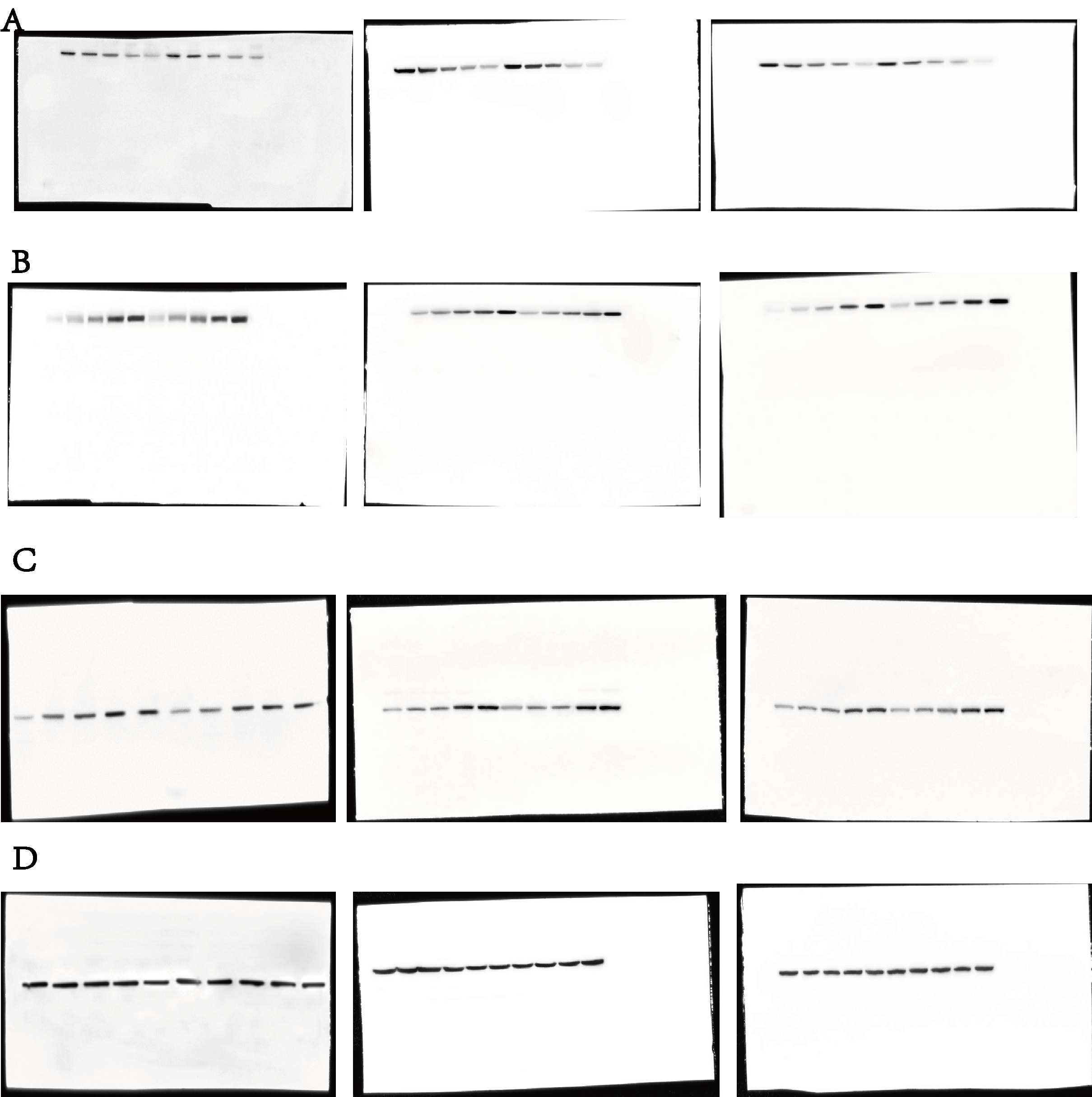
**

**Supplemental Figure 3**

**
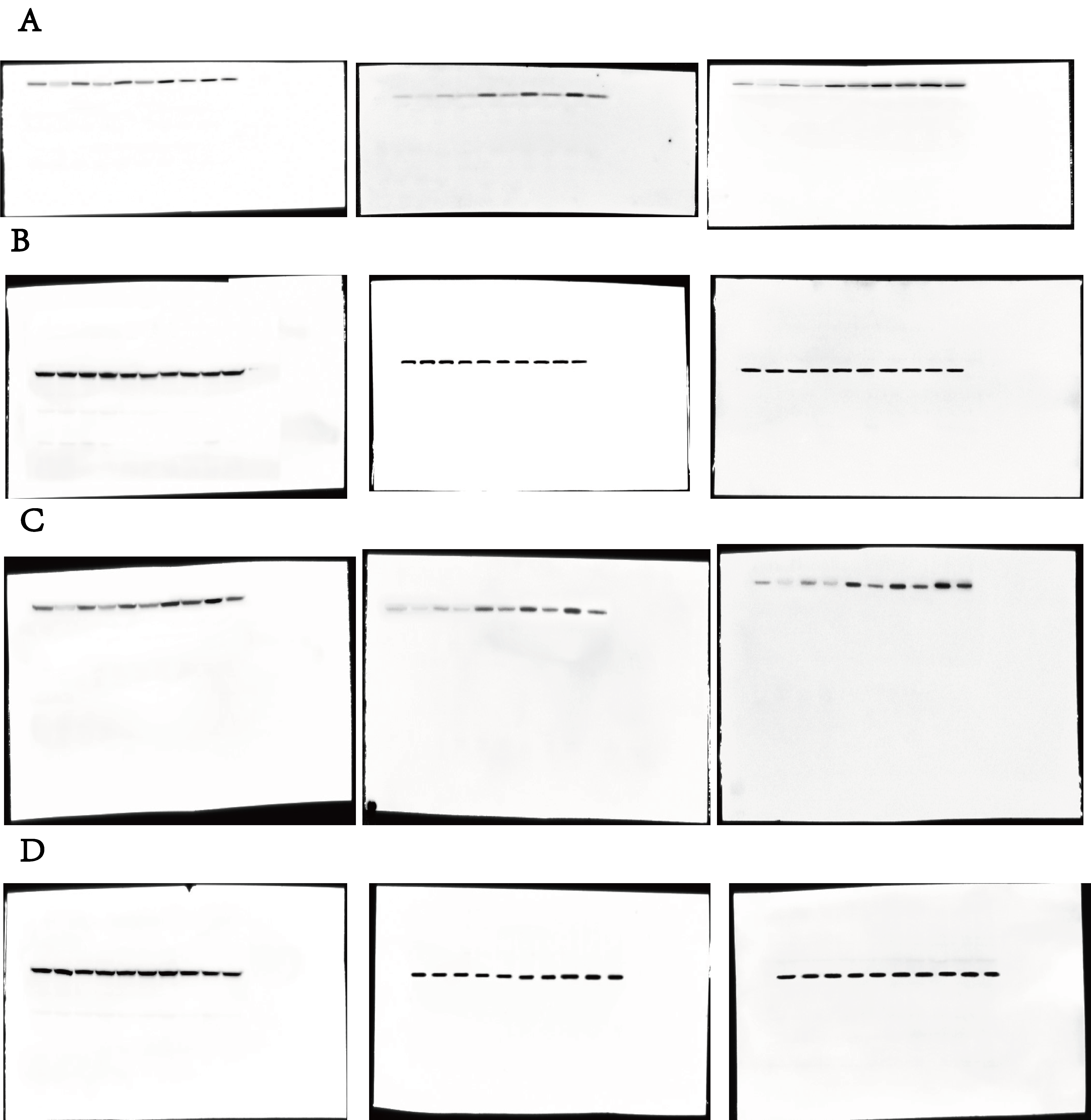
**

**Supplementary Figure 4**

**
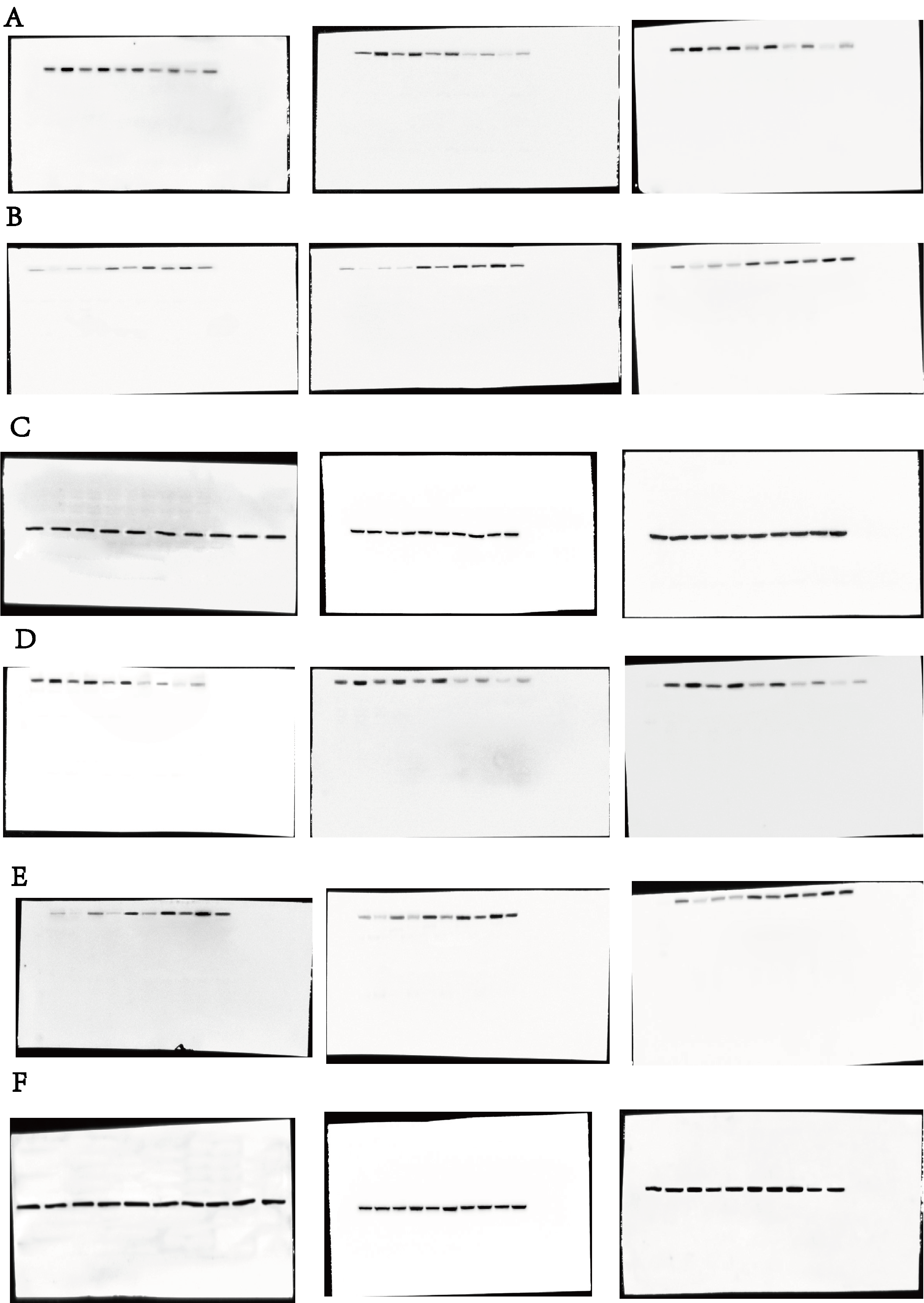
**

**Supplementary Figure 5**

**
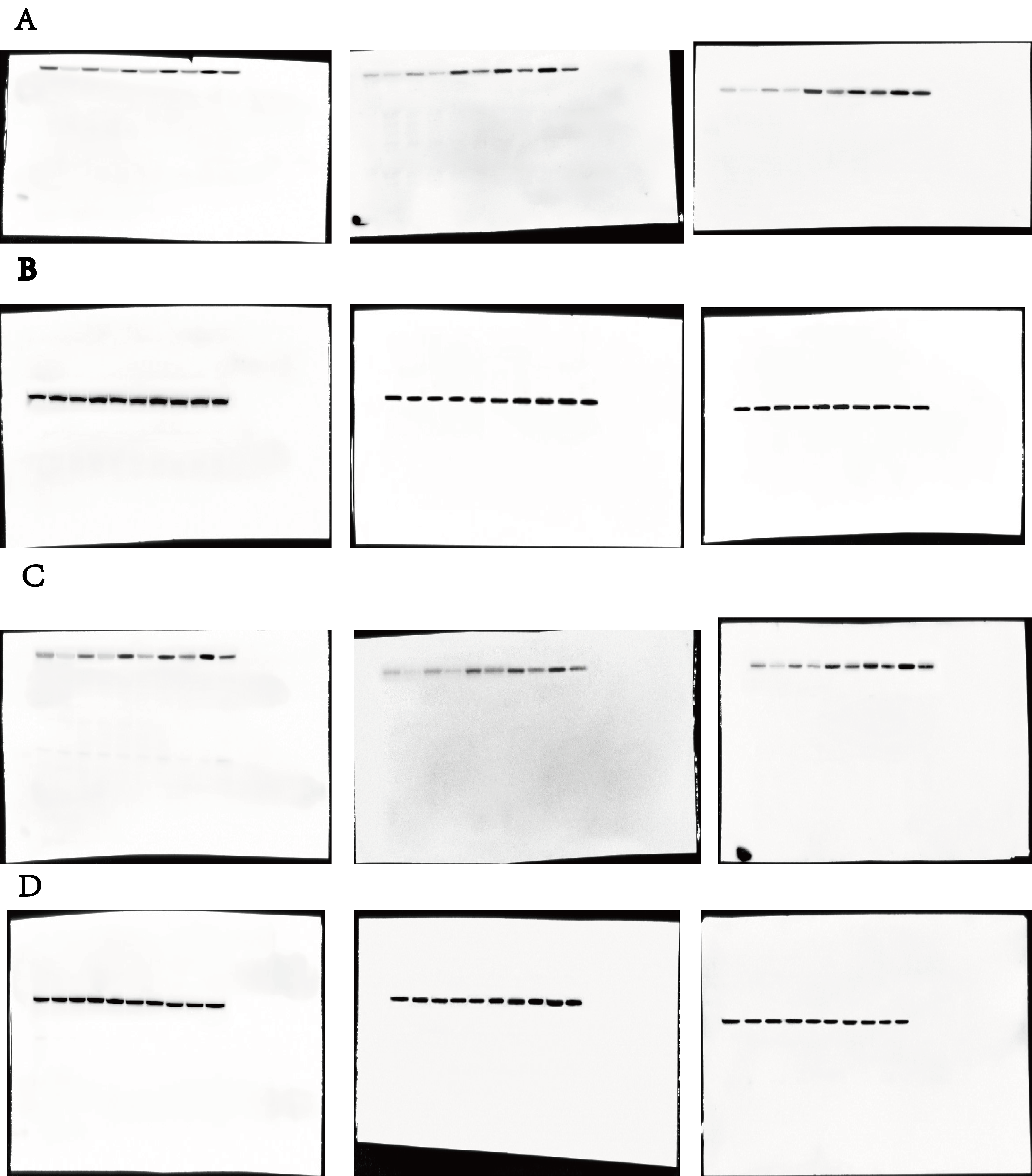
**

**Supplementary Figure 6**

**
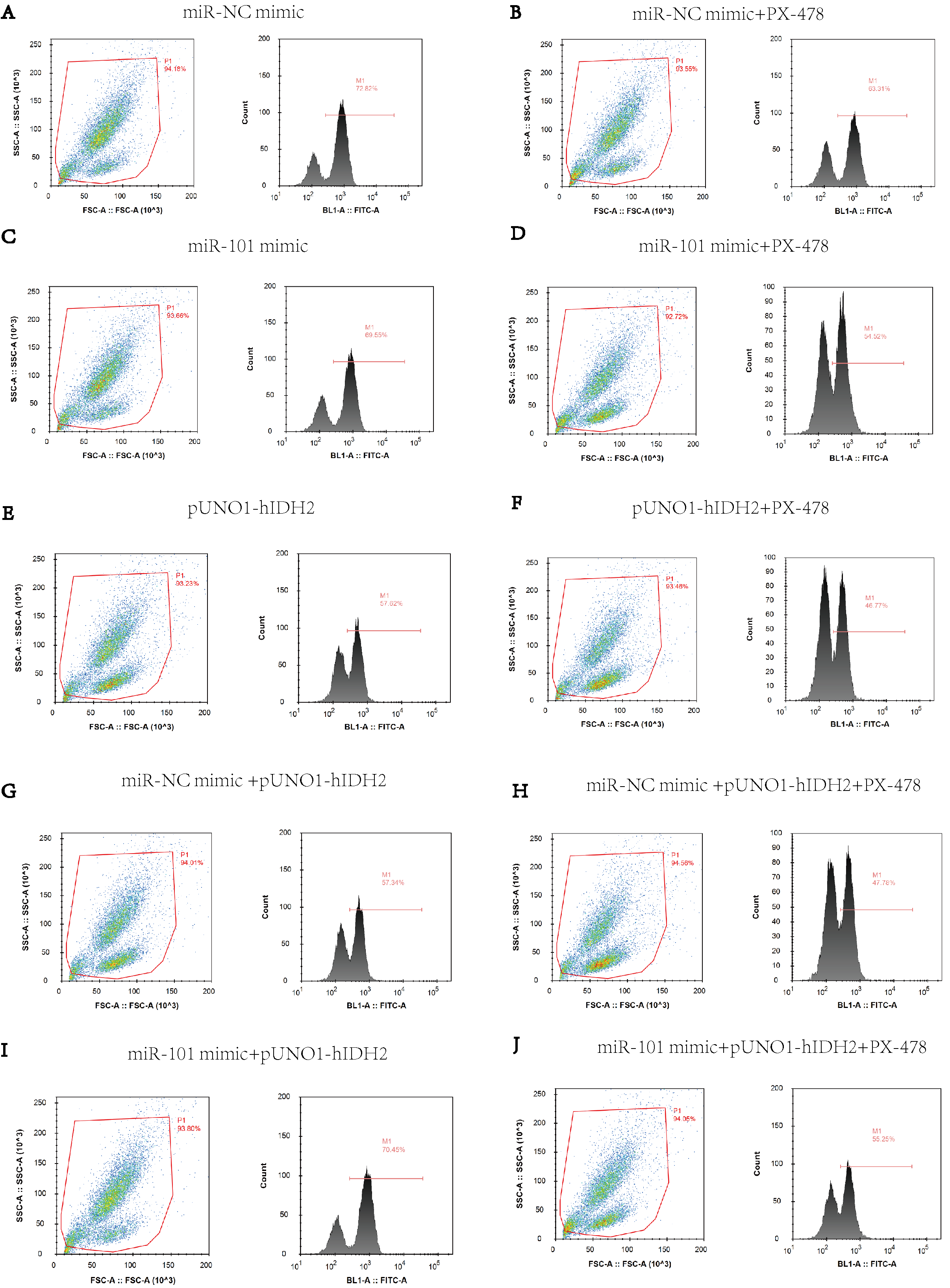
**

**Supplementary Figure 7**

**
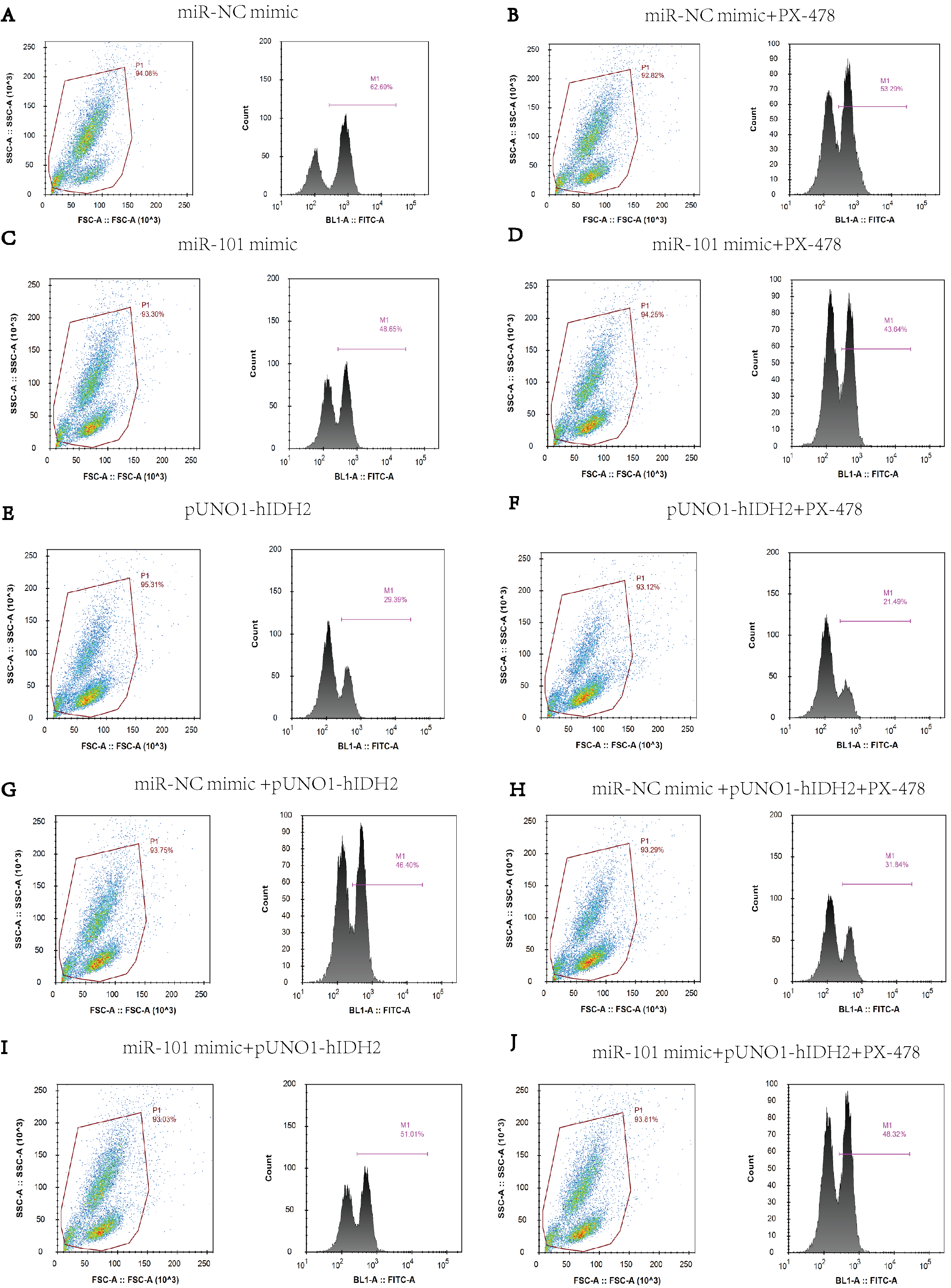
**

**Supplementary Figure 8**

**Supplementary Table 1.**

| Name | Gender | Age | Pathology | Stage |
| --- | --- | --- | --- | --- |
| Chunzhi Zhao | Male | 69 | Adenocarcinoma | IIIA |
| Xuesheng Han | Male | 60 | Adenocarcinoma | IA |
| Yuncai Li | Male | 74 | Squamous carcinoma | IIB |
| Hong Liu | Male | 71 | Adenocarcinoma | IIB |
| Haofu Xie | Male | 71 | Adenocarcinoma | IA |
| Humin Zhang | Male | 49 | Squamous carcinoma | IIB |
| Xiaoyan Zhang | Female | 67 | Adenocarcinoma | IB |
| Cailan Zhang | Female | 70 | Adenocarcinoma | IIIA |
| Yuting Chao | Male | 73 | Squamous carcinoma | IB |
| Yongming Lei | Male | 66 | Squamous carcinoma | IIB |
| Shuanyin Jia | Male | 64 | Adenocarcinoma | IB |
| Xiaoyin Liu | Female | 65 | Adenocarcinoma | IA |
| Xiaomei Meng | Female | 44 | Adenocarcinoma | IA |
| Caixue Zhu | Male | 74 | Squamous carcinoma | IIIA |
| Ailing Jin | Female | 58 | Adenocarcinoma | IIIA |
| Yaru Feng | Female | 63 | Adenocarcinoma | IIIA |
| Chengxin Yan | Male | 68 | Adenocarcinoma | IA |
| Desheng Wang | Male | 65 | Adenocarcinoma | IA |
| Genxiong Li | Male | 75 | Adenocarcinoma | IB |
| Gongxuan Liu | Male | 64 | Squamous carcinoma | IIB |
| Daiping Chen | Female | 64 | Adenocarcinoma | IIB |
| Jintang Liu | Male | 78 | Squamous carcinoma | IIIB |
| Xiaohong Li | Female | 58 | Adenocarcinoma | IIIA |
| Xuehong Tao | Female | 49 | Squamous carcinoma | IIIA |
| Yongcai Wang | Female | 52 | Adenocarcinoma | IIA |
| Yinlong Miao | Male | 69 | Squamous carcinoma | IIA |
| Qinsheng Wei | Male | 71 | Squamous carcinoma | IB |
| Xiping Liu | Female | 41 | Adenocarcinoma | IA |
| Baoli Hao | Male | 54 | Adenocarcinoma | IB |
| Fang Bai | Female | 58 | Adenocarcinoma | IA |
| Shuxian Zhang | Female | 77 | Adenocarcinoma | IIA |
| Zhoulinag Chang | Male | 54 | Squamous carcinoma | IIB |
| Yanling Luo | Male | 70 | Squamous carcinoma | IIA |
| Yong Shi | Male | 77 | Squamous carcinoma | IIIA |
| Xiaoli Bai | Female | 50 | Adenocarcinoma | IA |
| Runye Zhu | Female | 56 | Adenocarcinoma | IV |
| Xiangwu Wang | Male | 67 | Squamous carcinoma | IB |
| Qiaorong Wang | Female | 70 | Adenocarcinoma | IIA |
| Changxi Wu | Male | 65 | Squamous carcinoma | IIB |
| Gengshi Ren | Male | 64 | Squamous carcinoma | IIA |
| Caixia Ma | Female | 64 | Adenocarcinoma | IA |
| Fuding Lei | Male | 66 | Adenocarcinoma | IB |
| Yuchuan Cao | Male | 57 | Squamous carcinoma | IIIB |
| Yiyin Wang | Female | 67 | Adenocarcinoma | IA |
| Mangsheng Li | Male | 63 | Squamous carcinoma | IIA |
| Quanqi Xin | Male | 59 | Adenocarcinoma | IA |
| Yunying Wang | Female | 59 | Adenocarcinoma | IB |
| Qi Li | Male | 58 | Adenocarcinoma | IA |
| Zhengjiang Han | Male | 53 | Adenosquamous carcinoma | IIA |
| Jihe Wang | Male | 71 | Adenocarcinoma | IIIA |
